# Assessing the Zoantharian Diversity of the Tropical Eastern Pacific through an Integrative Approach

**DOI:** 10.1038/s41598-018-25086-4

**Published:** 2018-05-08

**Authors:** Karla B. Jaramillo, Miriam Reverter, Paul O. Guillen, Grace McCormack, Jenny Rodriguez, Frédéric Sinniger, Olivier P. Thomas

**Affiliations:** 1 Escuela Superior Politécnica del Litoral (ESPOL), Centro Nacional de Acuicultura e Investigaciones Marinas (CENAIM), Campus Gustavo Galindo Km. 30.5 Vía Perimetral, P.O. Box 09-01-5863, Guayaquil, Ecuador; 2 Zoology, School of Natural Science and Ryan Institute, National University of Ireland Galway (NUI Galway), University Road, H91 TK33 Galway, Ireland; 3 Marine Biodiscovery, School of Chemistry and Ryan Institute, National University of Ireland Galway (NUI Galway), University Road, H91 TK33 Galway, Ireland; 4 Tropical Biosphere Research Center, University of the Ryukyus, Sesoko Island, Okinawa, 905-0227 Japan

## Abstract

Zoantharians represent a group of marine invertebrates widely distributed from shallow waters to the deep sea. Despite a high diversity and abundance in the rocky reefs of the Pacific Ocean, very few studies have been reported on the diversity of this group in the Tropical Eastern Pacific coasts. While molecular techniques recently clarified some taxonomic relationships within the order, the taxonomy of zoantharians is still highly challenging due to a lack of clear morphological characters and confusing use of different data in previous studies. Our first insight into the zoantharian diversity at El Pelado Marine Protected Area - Ecuador led to the identification of six species: *Terrazoanthus patagonichus*; *Terrazoanthus* sp.; *Antipathozoanthus hickmani*; *Parazoanthus darwini*; *Zoanthus* cf. *pulchellus*; and *Zoanthus* cf. *sociatus*. A metabolomic approach using UHPLC-HRMS was proven to be very efficient as a complementary tool in the systematics of these species and specialized metabolites of the ecdysteroid and alkaloid families were identified as key biomarkers for interspecific discrimination. These results show good promise for an application of this integrative approach to other zoantharians.

## Introduction

The marine biodiversity of the Tropical Eastern Pacific ecoregion has been poorly studied in comparison to hotspots of biodiversity like the Central Indo-Pacific with a notable exception of the Galapagos islands^1^. Despite a shorter coastline than Chile, Peru or Colombia, Ecuador has a peculiar location in the Eastern Pacific for the study of the marine biodiversity due to oceanographic conditions largely influenced by seasonal oscillations of the equatorial front, which separates two important warm (Panama or El Niño) and cold (Humboldt) Pacific currents with frequent upwellings^2–4^. A national project has recently been funded with the main aims to describe the biological and associated chemical diversity in a specific mainland area of the Marine Protected Area El Pelado (REMAPE), Santa Elena, Ecuador.

From the large diversity of marine invertebrates present in this ecoregion, we dedicated our initial taxonomical and chemical efforts towards the order Zoantharia due to their high substrate cover, taxonomical diversity, and putative chemical diversity. Relatively few studies have been focused on the diversity of zoantharians in the Eastern Pacific since the first descriptions of zoantharians from Chile over a century ago: *Terrazoanthus patagonichus* by Carlgren 1898^5,6^, originally described as *Epizoanthus patagonichus*; and *Parazoanthus elongatus* by McMurrich, 1904^6^. In the last decade, additional studies have been conducted along the South American coasts of the Pacific revealing other zoantharian species and their distribution^7–10^. In the Tropical part of the Eastern Pacific, the first studies on zoantharian diversity were carried out in the Galapagos islands and they reported the presence of species from the genus *Terrazoanthus*, *Parazoanthus darwini* and *Antipathozoanthus hickmani*^8^. Despite a recent insight into the zoantharian diversity in the northern part of mainland Ecuador (Machalilla national park)^11^, the zoantharian diversity remains to be fully assessed over most of the Eastern Pacific and especially off the Ecuadorian coast^8^.

For decades, taxonomic descriptions of species in the order Zoantharia were based on comparisons of various morphological features such as number of tentacles, polyps/colonies measurements, color, shape and position of sphincter muscle and size and distribution of nematocysts^12–14^. Currently the main and widely accepted morphological character is the organization of the septa that distinguishes the two suborders, Macrocnemina and Brachycnemina, this distinction being further supported by differences in reproductive biology^15^. However, the interpretation of some other morphological characters to identity zoantharians at species level is still unclear because most of the diagnoses on type specimens are based on different characters rendering comparisons difficult^16^. Recent studies re-exploring microanatomy such as the sphincter muscle showed promising results, corroborating molecular data^17,18^. Yet, biased interpretations of the sphincter data has also led to taxonomic confusion^19^ and in addition to the high technical skills required and significant data interpretation^20^, it remains to be seen how this approach can be standardized among different researchers. Therefore, identification at species level remains a true challenge^21^ and molecular approaches have recently been applied to revise this complex order^16,18,22–25^. While this approach has become the main source of evidence supporting the discovery and description of higher taxa and species^18,24,26–28^, relationships between closely related species remain largely unresolved^25,29^. Given that the history of zoantharian taxonomy leaves many gaps, including poorly characterized taxa^13^ with inconsistent molecular and ecological data, and also discrepancies between morphological and DNA-based taxonomy^18,24,30–32^, we decided to add metabolomic data to shed light on this confused situation and contribute to the development of an integrative systematics of the order Zoantharia.

The biochemical information contained in the metabolite expression of living organisms have recently been proposed as a useful complementary tool in integrative systematics and was first applied to plants and microorganisms^33–37^. Marine invertebrates are also good candidates for the use of metabolic information as they are known to produce a large diversity of specialized metabolites also called natural products^38^. Unlike primary metabolites, these small organic molecules are not directly involved in the development of the organism, but their rather specific ecological roles make them good candidates in an integrative systematics approach. Indeed, from an evolutionary perspective, they may represent final products of biosynthetic pathways with key ecological roles such as defense or competition. Zoantharian specialized metabolites have mainly been investigated to identify new chemicals with applications as medicines for human health^39–41^. For instance, the structurally complex and extremely toxic palytoxin was isolated from a zoantharian of the genus *Palythoa*^42,43^. The usual natural product chemistry approach is clearly not satisfactory for systematics due to a skewed and more pharmaceutically driven process precluding the emergence of chemotaxonomy, and a broader vision of the metabolic content is required^44^. The recent development of environmental metabolomics based on Nuclear Magnetic Resonance (NMR) and Mass Spectrometry (MS) paved the way for a broader coverage of the metabolites produced by a single organism or cell in a short period of time and with high sensitivity, avoiding the long and tedious process of isolation and identification of the metabolites. Metabolomic approaches were recently applied to some groups of sponges and cnidarians providing valuable insights into their classifications^21,44–47^. For example, a HPLC-MS untargeted metabolomic approach applied to the classification of Mediterranean Homoscleromorpha sponges yielded data well correlated to phylogenetic analyses^46^. For zoantharians, a more targeted approach facilitated the identification of the different families of secondary metabolites responsible for the separation of two morphotypes of the Mediterranean *Parazoanthus axinellae*^21^. In the latter study, morphological and molecular data of both morphotypes of *P*. *axinellae* were consistent with only one species but clear differences in their metabolic profiles supported the existence of two distinct species. Conversely, in a recent study on the use of metabolomics to assess geographical *versus* specific metabolomic variability for two species of the genus *Palythoa* along the Brazilian coast, the authors observed a higher intraspecific variability comparing to the interspecific one, therefore questioning the use of metabolomics for the discrimination between zoantharian species^48^. Such contradictory results prompted us to investigate the potential of metabolomics as a complementary tool to morphological and molecular data in species identification of zoantharians collected in the REMAPE, Ecuador.

## Results

### Morphological identification

**Figure 1:**
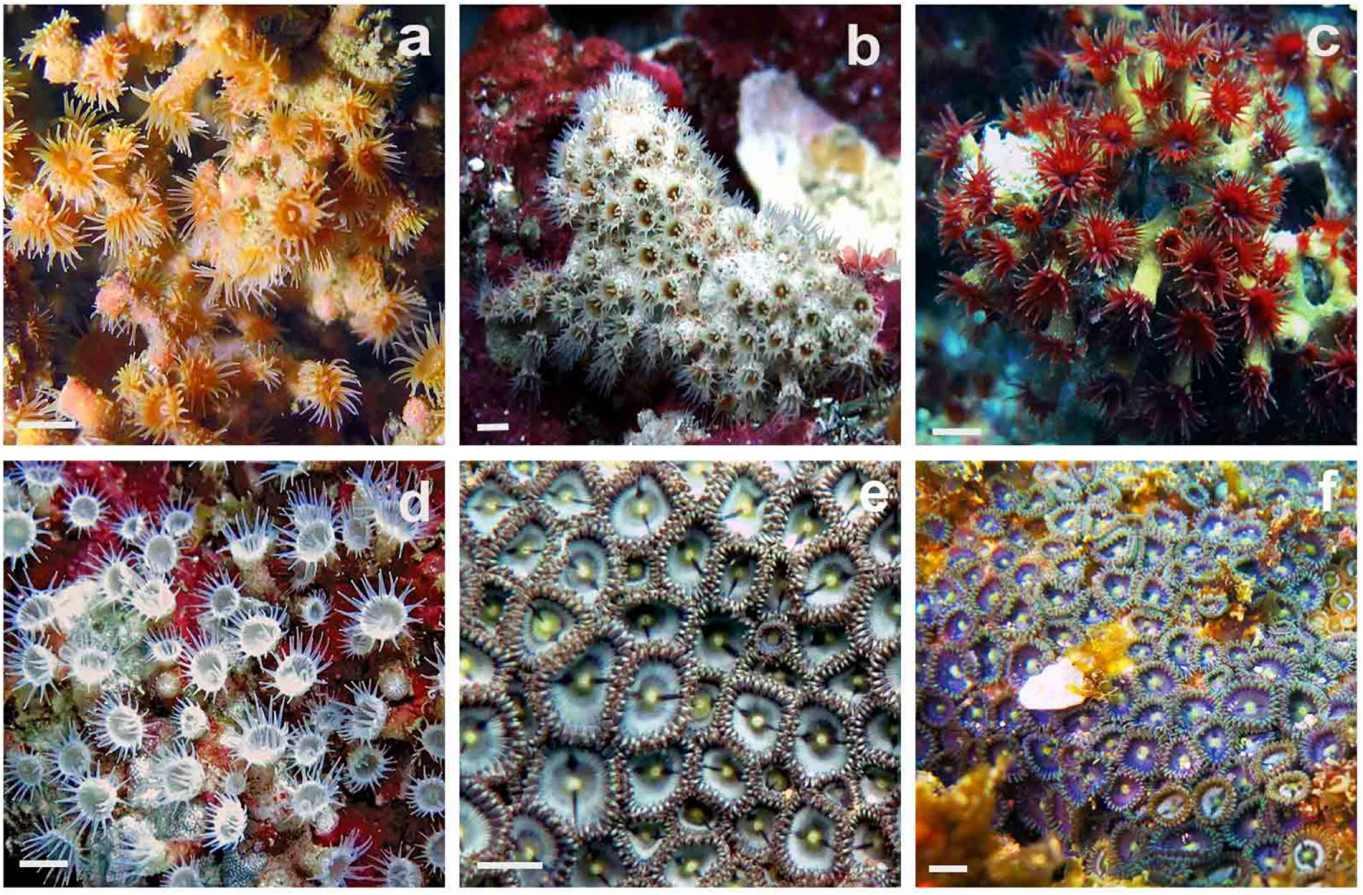
*In situ* images of the six zoantharians species discussed. (**a**) *Parazoanthus darwini* (Z1); (**b**) *Antipathozoanthus hickmani* (Z2); (**c**) *Terrazoanthus patagonichus* (Z3); (**d**) *Terrazoanthus* sp. (Z4); (**e**) *Zoanthus* cf. *pulchellus* (Z5); (**f**) *Zoanthus* cf. *sociatus* (Z6). The scale bar represents 1 cm. (Pictures from K.B. Jaramillo).

Based on morphological characters, six species of zoantharians (Z1 to Z6) belonging to three families (Hydrozoanthidae, Parazoanthidae, and Zoanthidae) and four genera (*Terrazoanthus, Parazoanthus, Antipathozoanthus*, and *Zoanthus*) were first proposed for the 12 specimens collected (Fig. 1). The morphological characteristics of the six zoantharian species are summarized in Table 1 and compared with literature data.

**Table 1:** Comparison of morphological and ecological characteristics of the species found at REMAPE with similar species described in previous studies. Species in bold were identified in this study. The word na = means not available.

Species/sample name	Polyp diameter alive/fixed (mm)	Tentacle number	Mesenteries number	Color (tentacle/oral disc/column)	Sphincter muscle	Substrate	Mineral incrustations
**Z1** ***Parazoanthus darwini***	**3–6/1–3**	**24–28**	**Na**	**Orange or cream**/**pale orange**/**light pink or cream**	**Cteniform endodermal** ^**a**^	**Rocks**/**small cracks**	**Heavily mineral particles incrusted**
*Parazoanthus darwini* ^8^	3–6/Na	24–30	Na	Yellow, orange or cream/red, yellow or light yellow/light tan, light pink or cream.	Mesogleal form (Cteniform endodermal)^a^	(Usually) sponges.	Polyps and coenenchyme incrusted with black sand.
**Z2** ***Antipathozoanthus hickmani***	**6–12/2–4**	**36–40**	**Na**	**Yellow or orange**/**Cream-yellow**/**orange-cream**.	**Cteniform endodermal** ^**a**^	**Branches of black corals (*Antipathes galapaguensis*** **and** ***Myriopathes panamensis*)**.	**Sand incrusted in the polyps and coenenchyme**.
*Antipathozoanthus hickmani* ^8^	6–12/Na	Approx. 40	Na	Bright yellow and/or red/Na/long red, cream or yellow.	Endodermal form (Cteniform endodermal)^a^	Branches of Antipathes galapaguensis.	Sand incrusted in polyps and coenenchyme.
**Z3** ***Terrazoanthus patagonichus***	**4–10/2–4**	**34–40**	**Na**	**Bright red with small bright white spots on the tips**/**dark red**/**yellowish or brown**.	**Meso-endo transitional** ^**a**^	**Rocks**/**black corals (*A***. ***galapaguensis*** **and** ***M***. ***panamensis*)**.	**Heavily incrusted by mineral**
*Terrazoanthus patagonichus* ^70^	Na/3–5	Na	32–36 (arranged in 16 pairs)	Rust red/Na/sandy grey or brownish yellow.	Mesogleal type (meso-endo transitional)^a^	Hydroids, rocks and shells.	Heavily incrusted large sand grains.
*Terrazoanthus onoi* ^8^	4–12/Na	32–40	Na	Bright red/bright red or red brown/tan to dark brown.	Meso-endo transitional^a^	Upper surfaces of rocks, non-living substrate.	Polyps and coenenchyme incrusted with sand.
*Epizoanthus elongatus* ^71^	Na/2–2.5	46	42–46	Na/in alcohol- dark yellowish brown beneath the sandy layer (differently colored grains).	Reticulate mesogleal^a^	Rocks.	Small grains of sand.
**Z4** ***Terrazoanthus*** **sp**.	**Approx**. **3–7/1–3**	**30–34**	**Na**	**Bright white**/**white**/**cream or light brown**.	**Meso-endo transitional** ^**a**^	**Rocks and small crevices**.	**Heavy sand incrustations in the column of the polyp**.
*Terrazoanthus sinnigeri* ^8^	Approx. 2–8/Na	30–36	Na	Transparent (when colored)/dull brown, white, or clear/light brown or grey with large particles.	Meso-endo transitional^a^	Underside of rocks, rubble, or dead shells, often in small cracks or crevices.	Polyps and coenenchyme incrusted with relatively large pieces of sand.
*Terrazoanthus californicus* ^72^	Na/3–4	36	34–38	Brown in alcohol (no details).	Meso-endo transitional^a^	Na	Sand, weak at the top of polyps.
**Z5** ***Zoanthus*** **cf**. ***pulchellus***	**Na/1–3**	**50–60**	**Na**	**Light to dark brown or olives**/**pale green**/**varied pale greenish or olives**.	Discontiguous mesogleal^a^	**Rocks and wave action, intertidal zones**.	—
*Zoanthus pulchellus* ^73^	3–4/Na	60–70	Na	Green tentacles/red inside and green at the boarder/Na.	Na	Reef zones.	—
*Zoanthus pulchellus* ^74^	6/Na*	60 approx.	26–28	Dark brown, green or olives/bright green with light radiating lines (internal mesenteries) green pale or yellow/pale and transparent.	Double mesogleal (Discontiguous mesogleal)^a^	Rocks and stones in shallow waters near the rocky parts coast areas.	—
*Zoanthus pulchellus* ^52^	4–6/Na*	50–60	Na	Na/green or yellow sometimes with pink, brown, or yellow patterning/Na.	Discontiguous mesogleal^a^	Rocks/shallow waters no intertidal zones.	—
*Zoanthus kuroshio* ^23^	6–12/approx. 3	50–64	42–52	Pale pink, light green or gray/pale pink varied/lighter pale purple (almost cream).	Discontiguous mesogleal^a^	Dead coral or rocks.	—
**Z6** ***Zoanthus*** **cf**. ***sociatus***	**Na/2–4**	**48–60 approx**.	**Na**	**Variable between purple, greenish or brown**/**purple or green**/**greenish**.	**Discontiguous mesogleal** ^**a**^	**Rocks of shallow waters and intertidal zones**.	—
*Zoanthus sociatus* ^75^	3–12/Na	50–60	Na	Na/variable usually green/variable, usually bluish-greenish.	Na	Rocks.	—
*Zoanthus sociatus* ^52^	5/Na	48–60	Na	Na/green, blue or yellow sometimes with patterning/Na.	Discontiguous mesogleal^a^	Shallow waters intertidal zones.	—
*Zoanthus sansibaricus* ^23^	3–12/1.8–4	48–53	48–53	Varied between individual colonies (orange, red, brown, green, purple, white, blue, yellow)/paler around oral disk/light to dark purple with no markings.	Discontiguous mesogleal^a^	Rocks and coral reefs areas with strong water currents and wave action.	—
*Zoanthus sansibaricus* ^51^	Na/3–4	48–50	44–48	Smoky brown with orange red spots on the inside/reddish brown with greenish radial stripes, gray white lips/gray body, whitish upwards.	Divided mesogleal (Discontiguous mesogleal)^a^	Reef rocks-Intertidal zones; the bodies are almost buried in the sand and only their heads stick out in the water.	—
*Zoanthus vietnamensis* ^76^	Na/3–5	Na	52–54	Grayish-green/pale-dark pink/grayish green	Divided mesogleal (Discontiguous mesogleal)^a^	Limestone, which is interspersed with drilling organisms Substrate.	—

**Na = **data not available.

^**a**^Evolutionary forms of the sphincter musculature, according to Swain 2015.

*Duerden 1898, mentions a considerable contraction of the polyps in ethanol.

The morphology of Z1 and Z2 corresponded to the descriptions of *Parazoanthus darwini* and *Antipathozoanthus hickmani* respectively from the suborder Macrocnemina while specimens assigned as Z3 and Z4 showed similarity to *Terrazoanthus* but were different from each other in their appearance. The tentacles of Z3 were bright red in color and the column was yellowish/brown, while Z4 had bright white tentacles/oral disk and a cream/light brown column. In addition, the diameter of the polyps of Z3 (4–10 mm) was usually larger than the diameter of the polyps of Z4 (3–7 mm). As such Z3 was consistent with *T*. *patagonichus* while Z4 was similar to *T*. *sinnigeri*. The morphological features of specimens of *Zoanthus* Z5 were clearly different from *Z*. *kuroshio* in terms of appearance and coenenchyme development. For *Z*. *kuroshio* the color of the polyps is pale pink and the tentacles are light pale pink, green or grey while, in Z5, polyps were pale green, and the tentacles were light to dark brown or olive (Table 1). *Z*. *kuroshio* also shows a well-developed, thick, rubbery, and lamellate coenenchyme while Z5 presented a laminar, smooth, and continuous one which adheres firmly to rocks and stones. These latter morphological features were very similar to the Caribbean *Z*. *pulchellus*. Specimens from Z6 showed similarities with the Atlantic *Z*. *sociatus* and *Z*. *sansibaricus* widely distributed in the Indo-Pacific. However, based on the number of tentacles and general appearance (color of the polyps, tentacles, and column), Z6 might be more related to *Z*. *sociatus* than *Z*. *sansibaricus*.

### Molecular analyses

The partial cytochrome oxidase subunit I (COI) was successfully sequenced for a total of 11 specimens and only samples of *A*. *hickmani* (Z2) could not be amplified for this marker (see Table 2 for details of specimens sequenced). The sequence of Z1-1 (807 bp) was identical to the recently described *P*. *darwini* (EU333788, EU333789, EU333795 all of 280 bp) and close to *P*. cf. *darwini* from Marquesas (MH013404, 782/783 identical bp) and *P*. *swiftii* (742/743 identical bp) from the Caribbean. Sequences from Z3-1 and Z4-1 to -2 were identical to each other and to the short sequences (280 bp) from the original descriptions of *T*. *onoi* (GU357557, EU333770, EU333774, EU333777 and EU333791-EU333794) and *T*. *sinnigeri* (GU357560, GU357567, GU357566) but they were slightly divergent (1 and 2 SNPs) from the longer sequences (593–595 bp) of *T*. *onoi* from mainland Ecuador (JN582016 and JN582917). Sequences of the four samples Z5-1 to -4 (all 806–807 bp) were identical to *Z*. *kuroshio* (KF499763, 497 bp and KF840073, 658 bp) but also to *Z*. *vietnamensis* (KF499749, 497 bp), *Z*. *pulchellus* (KT454374, 634 bp), *Z*. cf. *pulchellus* (KT454378, 634 bp) as well as to the shorter sequences from *Z*. *natalensis* (KJ416437 to KJ416439, all 244 bp). Sequences from Z6-1 to -3 (all 807 bp) were identical to *Z*. *sansibaricus* (KM267971, 643 bp; KM267980, 637 bp; KT426538, 566 bp), *Z*. cf. *sansibaricus* from Marquesas (MH013405) and *Z*. *sociatus* (EU348620, 658 bp and KT454370, 634 bp). The obtained phylogenetic tree, which corresponded to previous phylogenies using this marker, illustrated the lack of resolution between closely related species and placed each specimen into its expected clade (see Supplementary Fig. S1). Bootstrap support and posterior probabilities were high for deeper clades in the tree.

**Table 2:** Species, specimens, localities, voucher numbers and GenBank accession numbers analyzed in this study.

Species name	Sample Code	Locality	Depth (m)	Morphology	Metabolomics	COI	Mt 16S rRNA	ITS-rDNA	18S	Voucher Number
*Parazoanthus darwini*	Z1-1	Laberinto	10	✓	✓	MH029314	MH003893	—	MH029311	150825EP04-01
	Z1–2	Pared	25	✓	✓	—	MH003894	MH029324	—	150820EP01-05
	Z1–3	Laberinto	10	—	✓	—	MH003895	—	MH029313	150918EP04-24
	Z1–4	Acuario	12	—	✓	—	MH003896	—	MH029312	150918EP02-15
	Z1-5	Pared	22	—	✓	—	MH003897	—	—	160324EP01-10
	Z1-6	Pared	20	—	✓	—	—	—	—	160324EP01-11
*Antipathozoanthus hickmani*	Z2-1	Pared	26	✓	✓	—	MH003892	MH029334	—	150924EP01-02
	Z2-2	Pared	20	✓	✓	—	—	—	—	150820EP01-01
	Z2-3	Pared	24	—	✓	—	—	—	—	160324EP01-15
	Z2-4	Pared	25	—	✓	—	—	—	—	170317EP01-01
	Z2-5	Pared	26	—	✓	—	—	—	—	150813EP01-08
	Z2-6	Pared	28	—	✓	—	—	—	—	170810EP01-21
*Terrazoanthus patagonichus*	Z3-1	Pared	20	✓	✓	KY694966	KY694963	MH029328	MH029308	150820EP01-09
	Z3-2	Tello	31	—	✓	—	MH003898	MH029325	MH029309	150825EP03-03
	Z3-3	Pared	15	—	✓	—	—	MH029327	MH029310	150813EP01-11
	Z3-4	Pared	25	—	✓	—	—	MH029326	—	150813EP01-01
	Z3-5	Cuarenta	10	✓	✓	—	—	KY694964	KY694965	150807EP07-08
	Z3-6	Pared	10	—	✓	—	—	—	—	150820EP01-14
*Terrazoanthus sp*.	Z4-1	Laberinto	10	✓	✓	MH029316	MH003899	MH029329	MH029307	150918EP04-23
	Z4-2	Pared	15	✓	✓	MH029315	MH003900	MH029330	MH029306	170816AH01-05
	Z4-3	Laberinto	12	—	✓	—	—	—	—	160324EP04-01
	Z4-4	Laberinto	12	—	✓	—	—	—	—	160324EP04-02
	Z4-5	Laberinto	8	—	✓	—	—	—	—	160324EP04-03
	Z4-6	Laberinto	10	—	✓	—	—	—	—	160324EP04-04
*Zoanthus* cf. *pulchellus*	Z5-1	San Pedro	1	✓	✓	MH029320	MH003901	—	—	150819SP01-01
	Z5-2	Ayangue1	2	✓	✓	MH029321	MH003902	—	—	160505AY01-01
	Z5-3	Ayangue2	2	—	✓	MH029322	MH003903	—	—	160506FR01-02
	Z5-4	Ayangue1	2	—	✓	MH029323	MH003904	—	—	160506FR01-01
	Z5-5	Ayangue2	3	—	✓	—	—	—	—	160510AY02-01
	Z5-6	San Pedro	1	—	✓	—	—	—	—	160331SP01-01
*Zoanthus* cf. *sociatus*	Z6-1	Ayangue1	2	✓	✓	MH029317	MH003905	MH029331	—	151202AY01-01
	Z6-2	Ayangue2	4	—	✓	MH029318	MH003906	MH029332	—	160510AY02-03
	Z6-3	Ayangue1	1	✓	✓	MH029319	MH003907	MH029333	—	160505AY01-02
	Z6-4	Ayangue1	1	—	✓	—	—	—	—	160331AY01-01
	Z6-5	Ayangue1	1	—	✓	—	—	—	—	160331AY01-02
	Z6-6	Ayangue1	2	—	✓	—	—	—	—	160331AY01-03

**Figure 2:**
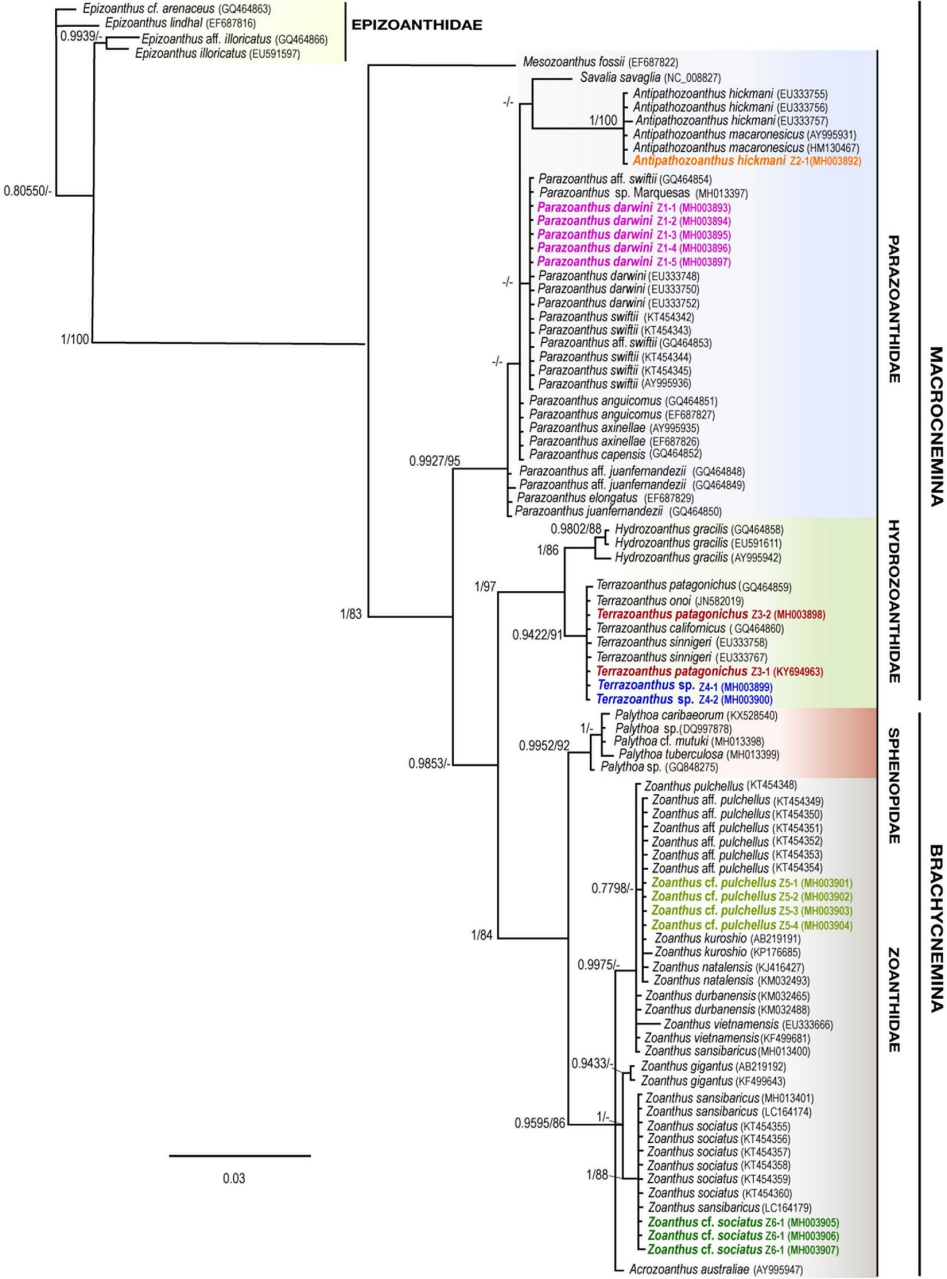
Phylogenetic Bayesian tree obtained from sequences of mitochondrial 16 S ribosomal DNA (mt 16 S rDNA). Bayesian and ML bootstrap support values over 0.75/75% are indicated by the nodes. Values below posterior probabilities of 0.75/75% bootstrap were considered as unresolved. Specimens from this study are indicated in different colors.

A total of 12 specimens were successfully sequenced for the mitochondrial 16S rDNA marker (Table 2). As also evident for CO1, Z1-1 to -4 sequences (all 1218 bp) were identical to recently described *P*. *darwini* (EU333748, EU333749 EU333750, EU333751 EU333752, EU333753, EU333754 all with 598 bp) and *P*. cf. *darwini* from Marquesas (MH013397, 1218 bp) but also *P*. *swiftii* from the Caribbean (AY995936.2, 1218 bp; KT454342–45, all 595 bp) and *P*. aff. *swiftii* (GQ464853 and GQ464854, 878 and 881 bp respectively). While the sequence from Z2-1 (1061 bp) was identical to most of the available sequences from the original descriptions of *A*. *hickmani* (EU333755, EU333756, both 582 bp) as well as the sequence from *A*. *macaronesicus* from East Atlantic (HM130467, 924 bp), but it was 1 bp different from the *A*. *hickmani* EU333757 sequence (582 bp). As for COI data, sequences from Z3-1 to -2 and Z4-1 to -2 were identical to sequences from the original description of *T*. *onoi* (EU333758-EU333764, and EU333766-EU333768, 561–573 bp). Compared to *T*. *patagonichus* (GQ464859, 871 bp) and *T*. *californicus* (GQ464860, 883 bp) and *T*. *onoi* (JN582019, 913 bp), sequences, all these specimens had one bp insertion in a poly C region. In addition, only the first bp of the sequence from *T*. *onoi* (JN582019) was different from the sequences obtained here, and this difference might be artifactual. The sequence from the original descriptions of *T*. *sinnigeri* (EU333765, 561 bp) showed two bp differences with other *Terrazoanthus* sequences. All three samples Z5-2 to -4 (all 879–881 bp) were identical to the sequences of *Z*. *kuroshio* from the original description (AB219191, 854 bp) and specimens from Taiwan (KF499671 and KF499673, 667 bp), as well as sequences from *Z*. aff. *pulchellus* from Brazil (KT454349, KT454340, KT454352-KT454354, 529 bp) and *Z*. *natalensis* (KJ416427 and KM032493, 426 and 445 bp respectively) but slightly different from another *Z*. *kuroshio* from India (KP176685, 846 bp) although these differences could be artifactual. All these sequences formed a subclade that was distinct from the *Z*. *pulchellus* sequence (KT454348, 529 bp). Sequences from specimens Z6-1 to -3 were identical to *Z*. *sociatus* sequences from Brazil (KT454356-KT454360, 529 bp) and differentiated by a single SNP from longer sequences of *Z*. *sansibaricus* (LC164174, 851 bp and LC164179, 861 bp) and *Z*. cf *sansibaricus* from Marquesas (MH, 770 bp). As for the previous marker, both ML and Bayesian trees showed similar topologies and corresponded to previously published phylogenies of the order (Fig. 2).

The Internal Transcribed Spacer region (ITS1, 5.8S and ITS2) was sequenced for a total of 11 specimens (Table 2). Compared to the sequences published in the original description of *P*. *darwini*, Z1-2 (780 bp) was identical to sequences EU333801 and EU333802 (635 bp), while sequences EU333799 and EU333800 (636 bp) were distinct by one SNP and one bp insertion. One SNP and a polyA stretch of 9 bp instead of 7 bp in ITS1 distinguished the sequence of Z2-1 (849 bp) from the original descriptions of *A*. *hickmani* (EU333797, 703 bp) and 5 additional SNPs within ITS1 separated the sequence of *A*. *hickmani* (EU333798, 703 bp). Sequences from some Z3 specimens (Z3-2 to -4, all 775 bp) were identical to *T*. *onoi* sequences (JN582023, 826 pb and EU333803, 625 bp) reported from mainland Ecuador^11^ and Galapagos, and separated by 1 SNP in ITS2 from most *T*. *onoi* from Galapagos (EU333804-EU333806, EU333808 and EU333809, 625 bp) and *T*. *patagonichus* from Peru (GQ464888, 831 bp). Sequences from the remaining Z3 specimens (Z3-1, Z3-5) but also from 2 Z4 specimens (Z4-1 to -2) differentiated by 2 SNPs in ITS2 from the previous group but were identical to *T*. *onoi* from mainland Ecuador (JN582022, 826 bp) and *T*. *californicus* from Peru (GQ464889, 831 bp). These latter sequences were distinct by 1 SNP at the end of the 5.8S rRNA from additional sequences of *T*. *onoi* from Galapagos (EU333807, 625 bp) and an additional 2 bp indel at the end of 18S (although the delimitation between 18S and ITS1 varied depending on the authors) from *T*. *sinnigeri* from the Galapagos (GU357553, GU357555, GU357556, 593 bp). Finally, sequences from three Z6 specimens (Z6-1, to -3, 815-821 bp) were all slightly different from each other and no corresponding sequences in the public database were found to match at the species level. However, these three sequences were most similar to the Atlantic species *Z*. *sociatus* (e.g. JX119132, 704 bp) and *Z*. *pulchellus* (e.g. EU418344, 893 bp) or the Indo-Pacific *Z*. *sansibaricus* “B” sensu^49^. These sequences are separated by numerous indels and SNPs from both *Z*. *natalensis* (e.g. KM032385, 377 bp) and most *Z*. *sansibaricus* (e.g. AB214136, 694 bp). Unfortunately, no unambiguous sequences could be obtained from *Zoanthus* cf. *pulchellus* (Z5-4).

The sequences obtained for the nuclear 18S rDNA represent the first sequences of this marker for zoantharian species from the South-Eastern Pacific. All the sequences from *P*. *darwini* were identical with one sequence from *P*. *swiftii* from the Atlantic (KC218417). Sequences from all the *Terrazoanthus* specimens were identical thereby not distinguishing between both species. 18S rDNA sequences could not be obtained from any of the *Zoanthus* specimens due to contamination with *Symbiodinium* zooxanthellae, and from *Antipathozoanthus* for unidentified reasons.

Combining the above morphological and molecular observations, we identified six zoantharian species as: Z1 *Parazoanthus darwini*; Z2 *Antipathozoanthus hickmani*; Z3 *Terrazoanthus patagonichus*; Z4 *Terrazoanthus* sp.; Z5 *Zoanthus* cf. *pulchellus*; Z6 *Zoanthus* cf. *sociatus*.

### Metabolomic analyses

**Figure 3:**
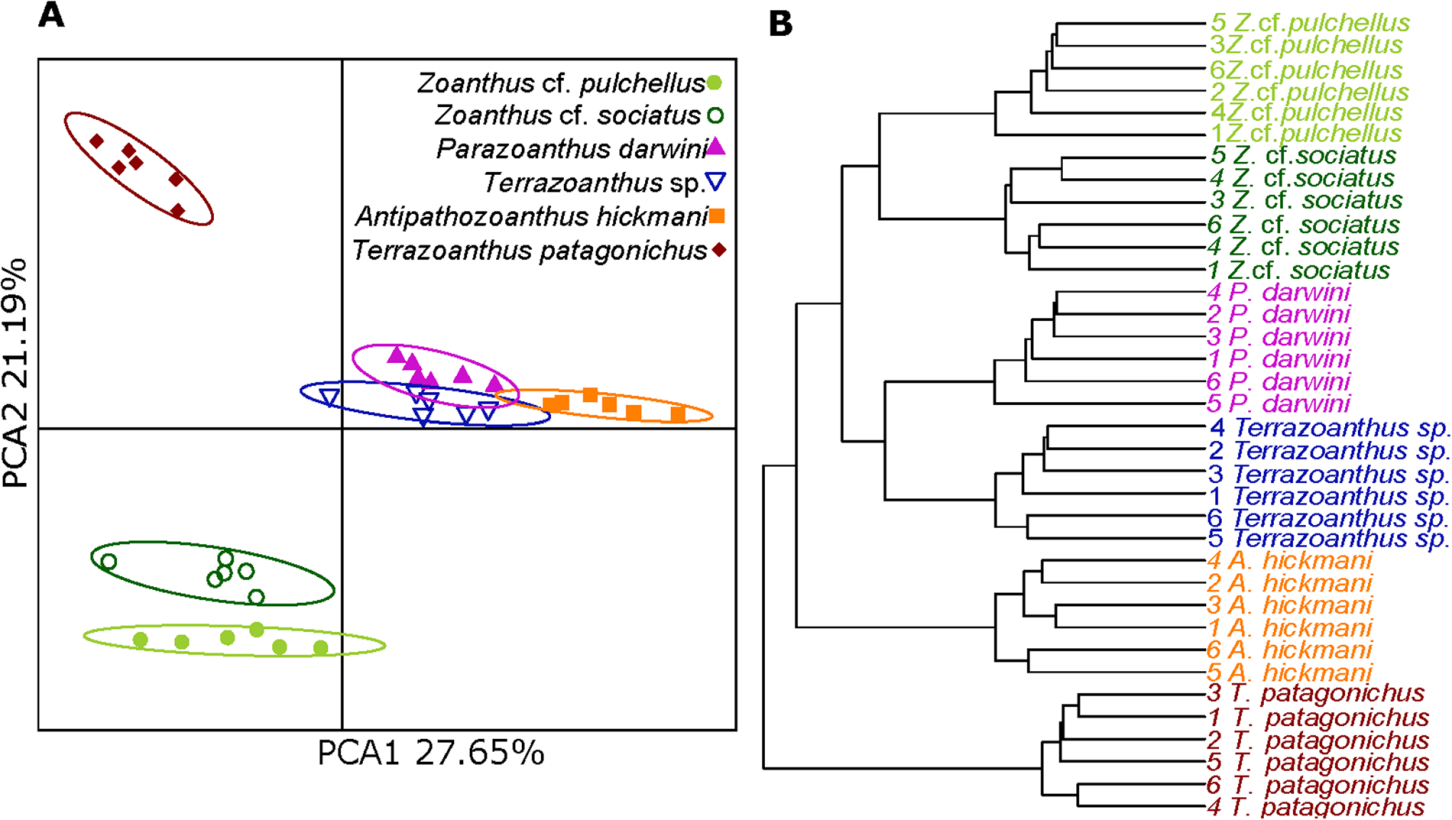
Untargeted Metabolomic Analysis of Zoantharians from this study. Score plots of metabolomic profiles of *Antipathozoanthus hickmani*, *Parazoanthus darwini*, *Terrazoanthus patagonichus*, *Terrazoanthus* sp., *Zoanthus* cf. *pulchellus*, and *Zoanthus* cf. *sociatus*. The explained variances are shown in brackets. (**A**) Principal Component Analysis. (**B**) Hierarchical Cluster Analysis.

A non-targeted metabolomic approach using UHPLC-HRMS was applied to 36 zoantharian specimens (six replicates for each species), including the 12 specimens used for morphological assessment and molecular studies. The metabolomic profiles were consistent between all replicates within a species and significantly different for the six-studied species (p < 0.01, Fig. 3A). Differences between species were emphasized when only the major metabolites (area ≥ 10^6^) were considered (p < 0.001, see Supplementary Fig. S2). A clear separation was for example observed between the two *Zoanthus* species when keeping only the major metabolites.

A hierarchical cluster analysis was then performed to investigate the similarity between the metabolomic profiles of the six zoantharian species. Each of the species formed a separate cluster, *T*. *patagonichus* being the species with the most distinct metabolome, followed by *A*. *hickmani*. The clusters of *P*. *darwini* and *Terrazoanthus sp*. were the most similar, followed closely by the two clusters of *Zoanthu*s species (Fig. 3B).

**Figure 4:**
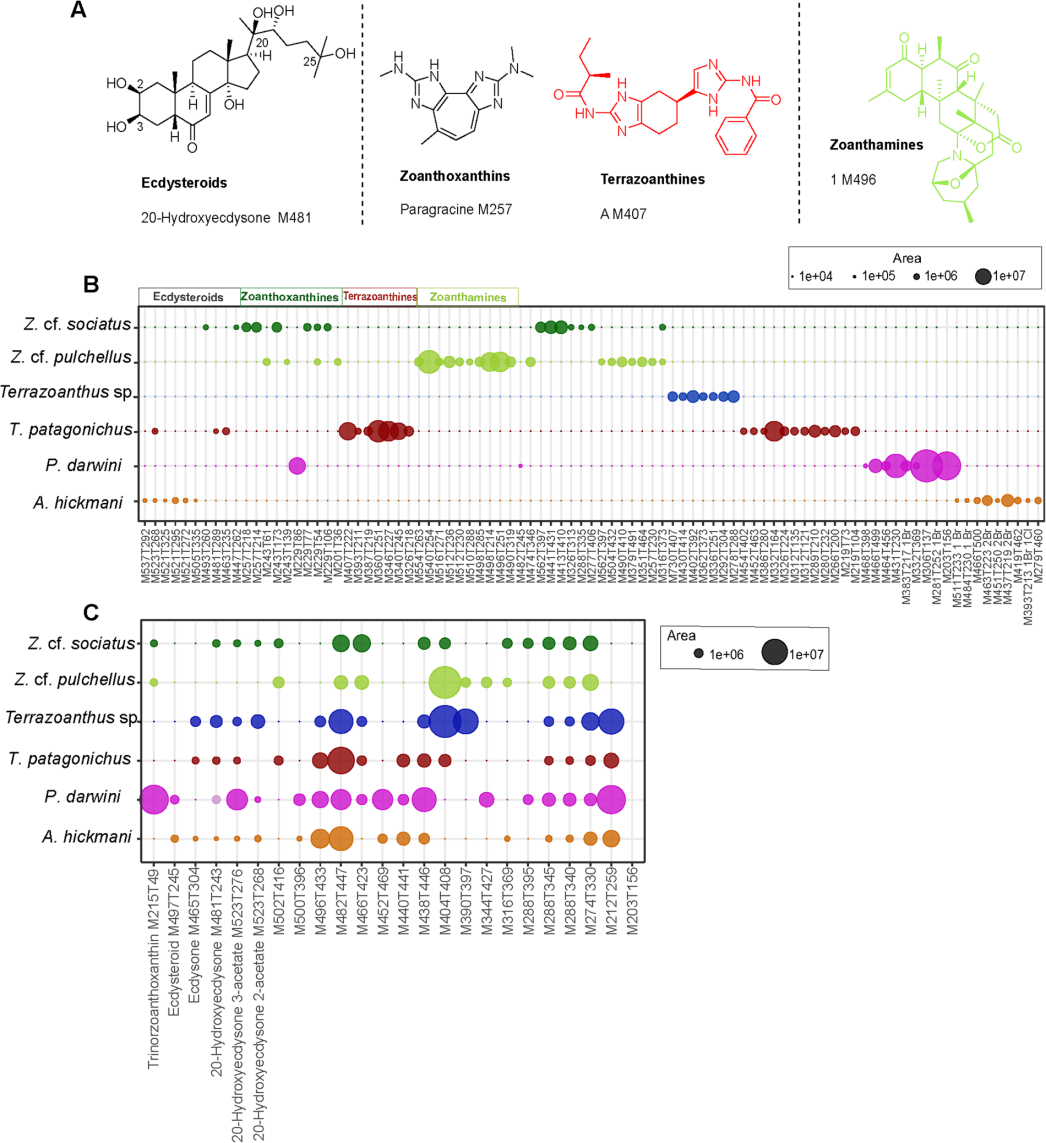
(**A**) Chemical structure of the major metabolite markers (areas ≥ 10^6^) of the six zoantharian species studied. (**B**) Main variables found in only one species of zoantharian. (**C)** Main variables found in more than one species of zoantharian. For each variable M = molecular weight, T = retention time. Different colors represent the six different zoantharians species.

A total of 113 major metabolites with areas higher than 10^6^ were observed from the six species of zoantharians. From them, 45 were identified as belonging to the following three families of natural products using available standards and unambiguous comparison with literature data: ecdysteroids due to their typical fragmentation pattern (loss of water molecules), zoanthoxanthins and related terrazoanthines (bisguanidinylated aromatic alkaloids or BGA), and zoanthamines (non-aromatic alkaloids) (Fig. 4A). Among the major metabolites, 89 were identified as markers of the different zoantharian species (Kruskal-Wallis and post-hoc Kruskal-Wallis, p-value < 0.001). Zoanthoxanthins were mostly found in *Z*. cf. *sociatus* but also in most species as minor metabolites (Fig. 4B). Absent in *T*. *patagonichus*, these BGA seem to be substituted in this species by a closely related and recently described family of alkaloids, the terrazoanthines^50^. The zoanthamines were only found in our species of *Z*. cf. *pulchellus*, while two other and unknown families of halogenated families were specifically found in *P*. *darwini* and *A*. *hickmani*. Finally, the species *Terrazoanthus* sp. was characterized by a range of unique and unknown metabolites with even values of mass over charge ratio (*m/z*).

Several ecdysteroids including ecdysone, 20-hydroxyecdysone, 20-hydroxyecdysone 2-acetate and 20-hydroxyecdysone 3-acetate were found in all the species but *Z*. cf. *pulchellus* (Fig. 4C). Very polar zoanthoxanthins were also found in most of the zoantharians species sampled here, while other unknown metabolites (sometimes with a strong intensity like M482T447) were also shared by several species. For each of these latter metabolites, we were unable to propose a structure and subsequent isolation work is needed to characterize them.

## Discussion

### Zoantharian Systematics

Our results, together with the most recent studies from Galapagos Islands^7–9^, revealed the unique diversity and abundance of zoantharians encountered in the Tropical Eastern Pacific ecoregion. Using a multidisciplinary approach, we identified a high diversity of zoantharians in a small area off mainland Ecuador with six species present from intertidal to deeper waters (30 m), including three potentially new species.

Our results well illustrate the challenges in zoantharian taxonomy, i.e. low variability in molecular data coupled with morphological distinctiveness and chemical diversity. For example, amongst representatives of the suborder Brachycnemina collected here, the mitochondrial markers COI and 16S confirmed the presence of two distinct *Zoanthus* species (Z5 and Z6), while being identical to species sequenced from other studies: Z5 to *Z*. *kuroshio* and *Z*. *pulchellus*, among others; and Z6 to *Z*. *sansibaricus* and *Z*. *sociatus*. The morphological characters suggested that our Z5 species is closely related to *Z*. *pulchellus* and Z6 more similar to *Z*. *sociatus* than *Z*. *sansibaricus*. However, direct comparisons with Atlantic specimens are needed to confirm the systematic relationships between Ecuadorian *Zoanthus* and Atlantic species.

In the suborder Macrocnemina, systematic data obtained for the two species of the family Parazoanthidae, *P*. *darwini* (Z1) and *A*. *hickmani* (Z2) were consistent with literature data. The minor inconsistencies in the DNA data from the ITS region of Z2 were interpreted as intraspecific variation. Species Z3 and Z4 were assigned to the recently described genus *Terrazoanthus* (Hydrozoanthidae family)^43^. On the basis of morphological characters and molecular data described here and in the literature, we consider *T*. *onoi* as a junior synonym of *T*. *patagonichus* and thus the latter is given priority^17^. Species Z4 showed clear morphological differences when compared to Z3. Thus, the low divergence of the molecular markers between Z3 and Z4 illustrates the difficulty in separating species of this genus based on DNA data. Despite identical COI and 16S rDNA sequences and similar ITS regions with *T*. *sinnigeri* and *T*. *californicus*, the lack of morphological and molecular data for *T*. *californicus* did not allow us to reach conclusions for Z4. However, Z4 is morphologically distinct from *T*. *sinnigeri* and they occupy different habitats with *T*. *sinnigeri* found under rocks or dead shells and sometimes in small cracks or crevices^8^ while specimens of Z4 were always found on exposed rocks or small crevices. Overall, the exact relationship between Z4, *T*. *sinnigeri* and *T*. *californicus* remains to be more deeply investigated.

Because the morphological characterization of species from the genus *Zoanthus* is known to be complex due to the presence of different morphotypes^23,51^, the comparison of morphological data obtained in this study with other reported species from the Pacific, Atlantic and Caribbean regions was not fully conclusive. The similarities in morphological and molecular data between Eastern Pacific zoantharians and the Caribbean *P*. *swiftii*, *Z*. *sociatus* and *Z*. *pulchellus* suggest strong affinities between the Caribbean and the Eastern Pacific zoantharians^52^. A distribution of the same species across the Panama Isthmus would imply either connectivity through transport of larvae in ballast waters or resistance of the larvae to the low salinity water of the Panama isthmus. Zoantharian larvae are known to disperse potentially far through oceanic currents^15,53^, and their survival in ballast waters or their tolerance to low salinity remains unknown. Recent examination of genetic data from zooxanthellae associated with the presumably rare Eastern Pacific scleractinian coral *Siderastrea glynni* suggest that, rather than an endemic rare species this coral is actually the Atlantic *S*. *siderea* and was likely introduced to the Eastern Pacific through the Panama isthmus^54,55^. Although we cannot currently and objectively affirm that our *Zoanthus* specimens do not belong to the Atlantic species, it is more likely that the Ecuadorian zoantharian fauna shares a common history with tropical Atlantic fauna followed by allopatric speciation and potential mixing with the tropical Pacific fauna. This hypothesis, referred as the vicariance hypothesis has been evoked for scleractinian corals based on fossil data but was never demonstrated on living coral, although it could not be rejected neither^56^. Therefore, deeper taxonomic examination and broader geographical sampling of these zoantharians in adjacent ecoregions (both Pacific and Atlantic) are needed before any conclusion can be given on the species identity and origin of the Ecuadorian zoantharians.

The systematics analyses performed herein were based on four molecular markers (mitochondrial 16S, COI and nuclear ITS-2, 18S) and external morphological characters. They showed a good separation at genus level and a good agreement with DNA sequences and morphological features reported from the original species descriptions. It is worth noting however that molecular markers did not allow clear distinctions between closely related species of the genera *Terrazoanthus* and *Zoanthus*, confirming a high level of conservatism in zoantharian DNA. Furthermore, the lack of available DNA sequences in GenBank for some genera such as *Terrazoanthus* and *Antipathozoanthus* limits a thorough comparison of species and the identification at species level remains a key challenge for these genera. The difficulties associated with the usual systematic approach as a combination of only morphological and molecular data strongly support the need for additional data to yield a more robust classification within this order.

### Metabolomics

In recent years, metabolomics has been recognized as a useful complementary tool for the classification of some plants but also marine invertebrate groups^44,46^. As discussed above, the taxonomy of zoantharians is a challenge, especially at the species level, and we therefore decided to use UHPLC-HRMS metabolic fingerprinting to see whether these data could clarify some taxonomic uncertainties. The rationale behind the use of this approach was to develop a method which would first allow a detection of most of the known specialized metabolites produced by the targeted species. Indeed, the expression of metabolic pathways by the holobiont (host and symbionts) might represent key data of high taxonomic relevance and therefore a complementary tool to the systematics analyses used for the classification. As previously mentioned in a recent article, we decided to name this approach phylometabolomics^57^. We anticipated that zoantharians represent an ideal group for a phylometabolomic study as the metabolites identified by usual natural product chemistry belong mainly to two families of well-known natural products: the ecdysteroids and the BGA. As exemplified in a recent work on the Mediterranean *P*. *axinellae*^21^, these major metabolites are easily ionized in MS and therefore clearly detected when present in a species. This information should represent a prerequisite before starting a phylometabolomic analysis. While some symbiotic prokaryotes have been suggested to produce many of the metabolites found in marine invertebrates, the high concentration of these targeted major metabolites may indicate contribution of the zoantharian itself and its genetic information.

The non-targeted approach applied here on Ecuadorian zoantharians clearly showed a high specificity of the metabolome. Furthermore, the major compounds present in some studied specimens were clearly represented by major peaks of high intensity. As anticipated, when we applied a filter on features with areas higher than 10^6^ the separation between the species was enhanced. This is a clear demonstration that minor metabolites are more closely linked to the environment than to species phylogeny and that the metabolomic analysis is more relevant for Zoantharia when considering the major components. From a phylogenetic perspective, the metabolomic cluster analysis was more consistent with phylogeny in the closer association of the two *Zoanthus* species (Suborder Brachycnemina) with each other than to the remaining four species (Suborder Macrocnemina). However, within the Macrocnemina, relationships are not as expected with the position of *T*. *patagonichus* making the suborder paraphyletic and the position of *Terrazoanthus* sp. rendering the family Parazoanthidae paraphyletic. Clearly this approach has great potential in species discrimination but its use in supplying synapomorphies for phylogenetic reconstruction still needs caution.

In a recent article on a large-scale study on the red alga *Asparagopsis taxiformis*, the metabolomic dendogram did not fit with the different lineages but was more related to environmental variations^58^. The explanation proposed in the article was the difficulty associated with the ionisation of the halogenated metabolites found as major metabolites in this alga. Recently, metabolomic analyses of two *Palythoa* species along the Brazilian coast showed high intraspecific metabolomic variability with geography^48^. It is likely that when extended to a broader region the species included in our study would also show more variability in their metabolomic patterns. However, in the case of the *Palythoa* study the authors did not give any evidence underlying species identity of the specimens collected and some genetic and morphological variability might have been observed across this large geographic scale. We believe that without morphological and molecular evidence supporting the identity of the specimens collected, it is not realistic to provide any reliable conclusion on the interpretation of metabolomic variability for taxonomy. As commented in a key opinion paper for natural product chemists published recently^59^, emphasis must first be given to describe the taxonomy when analyzing an organism for its content in specialized metabolites. Being conscious of these requirements we have included a precise taxonomic description of all collected specimens before starting our metabolomic analyses. Given the different metabolomic approaches used, it is also likely that minor metabolites were included in the Brazilian study masking major signal of higher taxonomic relevance^48^.

The lack of full correlation between metabolomic data and molecular phylogenies coupled with the possibility of intraspecific variability over large geographical distances do indicate some limits of the phylometabolomics approach, mainly due to the lack of correlations between specific biosynthetic pathways and mass spec data. Combining metabolic pathways data and phylogenetic analyses would be very informative for systematics and this approach called phylometabolic analysis has been applied recently to some groups of microbes^60^. However, this approach requires deep knowledge of metabolic pathways of the organism which is not yet the case for marine invertebrates, mainly because of the complex composition of the holobiont. In the meantime, a targeted phylometabolomic study would allow grouping of species expressing identical or similar metabolic pathways inherited from a common ancestor. We therefore need to build on and extend databases of metabolites like in the GNPS initiative. In the case of zoantharians this database should include at least four families of compounds: ecdysteroids, zoanthoxanthins, zoanthamines and halogenated derivatives.

Among these known features of the metabolomic analyses, ecdysteroids were found in five out of the six species of zoantharians, the most common being 20-hydroxyecdysone and its acetyl derivatives at *O*-2 and *O*-3. In the last species *Z*. cf. *pulchellus*, these compounds were also found but as minor metabolites, which may be due to the high concentrations and ionisation of zoanthamines alkaloids, the major metabolites of this species. While ecdysteroids have been reported occasionally from other marine invertebrates like sponges, their true origin could be small zoantharians present as epibionts^61^. With few exceptions, we therefore propose that ecdysteroids may be considered as markers of zoantharians in the marine environment.

The second targeted family of natural products, the BGA, were easily identified by a combination of MS and UV data for these very easily ionized guanidine alkaloids. Zoanthoxanthins, a well-known family of fluorescent BGA, are highly expressed in both *Zoanthus* species and some of them are also found in *P*. *darwini*. This result is consistent with the finding of derivatives of this family in the Mediterranean *P*. *axinellae*^21^. While this family of compounds is absent from the two *Terrazoanthus* species Z3 and Z4, we identified acylated BGA analogues named terrazoanthines in *T*. *patagonichus*. The only difference between terrazoanthines and zoanthoxantins being the type of Diels-Alder reaction ([4 + 2] or [6 + 4]) involved in the cyclisation/dimerization key step, acylation of BGA seems restricted to the *Terrazoanthus* genus. Finally, the only species where BGA was found absent is *A*. *hickmani*. The chemical study on this species has recently been published and does not mention any compound of the BGA family^62^. Therefore, BGA might also be present in most zoantharians except in species of the genus *Antipathozoanthus*, only found as epibionts, but additional studies are needed to confirm this assumption.

All species shared other metabolites usually with even *m/z* and either these variables are taxonomic markers of all six zoantharians or most probably they are easily ionized alkaloids (odd number of nitrogen) present in the environment that may not have any taxonomic relevance.

Aside from ecdysteroids and BGA, the other major features were much more specific to each species. Several families of compounds seem restricted to only one species and not to a genus. Interestingly, *Z*. cf. *pulchellus* is the only species that contains a high concentration and diversity of unique alkaloids called zoanthamines. The metabolomics profile of *Z*. cf. *sociatus* showed other unique but unknown metabolites. Both species of the genus *Zoanthus* (Z5 and Z6) only share two or three major metabolites, so that the similarity in their metabolomic profiles is due to non-major metabolites. This result could be explained by the expression of other common metabolic pathways of less easily ionized molecules or families of compounds present at lower concentrations, with a contribution of the associated microbiota. Interestingly, *Zoanthus* belongs to the suborder Brachycnemina characterized by an association with *Symbiodinium* dinoflagellates. These dinoflagellates may therefore be responsible for the production of common metabolites that bring together species of this group. Importantly, only Z5 (*Z*. cf. *pulchellus*) would contain genes responsible for producing the bioactive zoanthamines, therefore confirming a strong difference between these two species even if their morphology and habitat were similar.

In this work we report a cautious use of metabolomic data on six Ecuadorian species within an integrative approach. When comparing metabolomics with molecular data obtained with four molecular markers, we obtain clear separation between the species using specialized metabolites, with distinct families of metabolites present for each zoantharian species. For instance, *Terrazoanthus* (Z3 and Z4) and *Zoanthus* species (Z5 and Z6) were difficult to separate at species level with DNA markers while they were easily distinguished by their main metabolites. However, distances between species on the dendrogram generated from metabolomic data do not necessarily represent phylogenetic distance given that they represent a binary analysis of the expression of related metabolic pathways rather than a phylogenetic analysis of inherited traits. Molecular networking is another tool that is available today to identify families of natural products, but this approach can also be misleading as fragmentation patterns may differ between closely related compounds.

The results of metabolomics analyses performed on six species from the Tropical Eastern Pacific confirms the use of a more targeted analysis as a complementary tool for the systematics of zoantharians, especially for the difference between two species when molecular markers are not sufficient. We therefore recommend the inclusion of metabolomic data for future integrative taxonomy studies of zoantharians due to the presence of easily ionized major specialized metabolites. This rapid and reproducible method will provide valuable information for the classification of Zoantharia in an evolutionary perspective. We will now extend our approach to other Atlantic, Pacific and the Caribbean species to investigate the application of this targeted phylometabolomic approach in the classification of zoantharians at a broader scale.

## Materials and Methods

### Study sites

**Figure 5:**
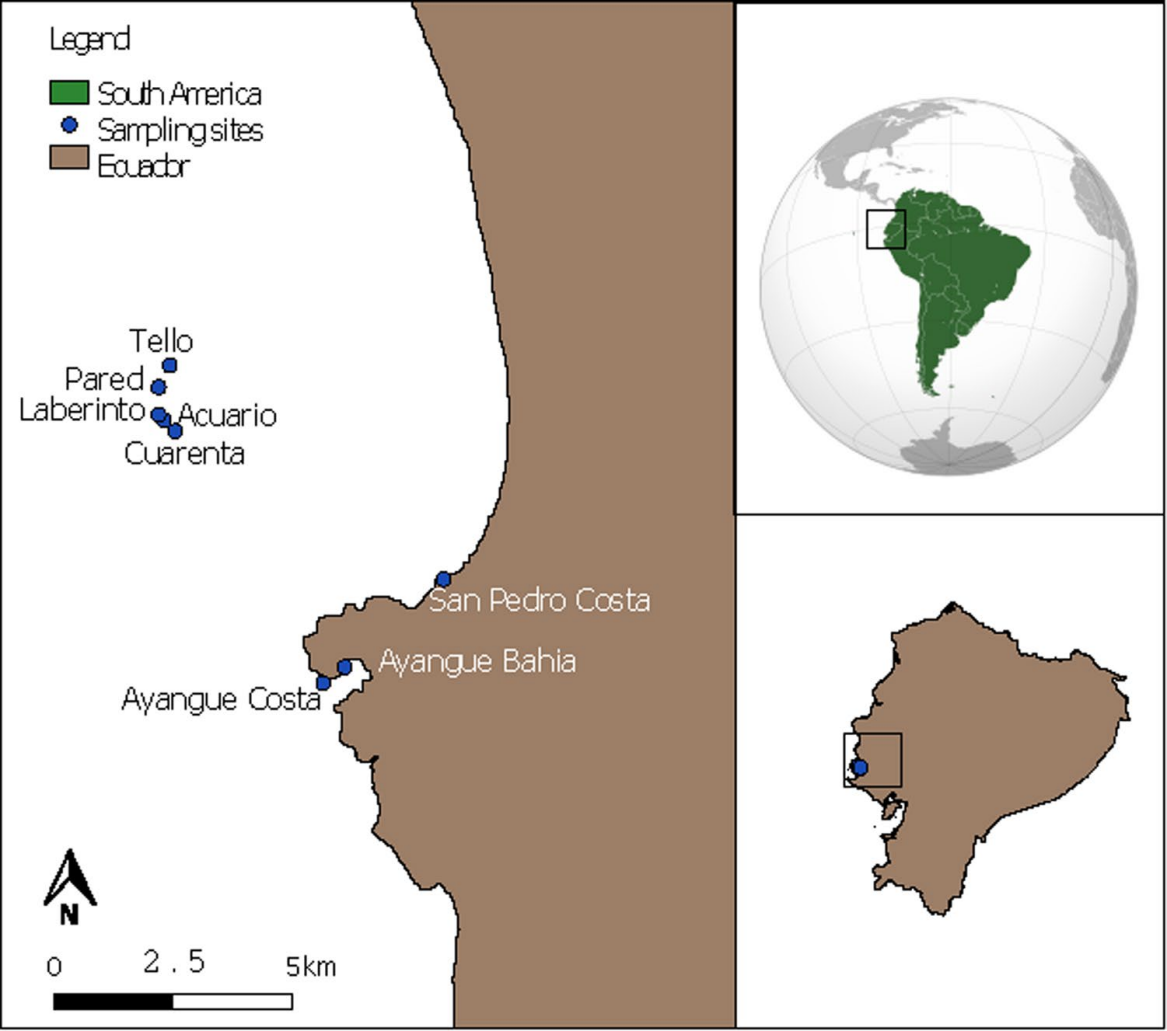
Map of the Marine Protected Area El Pelado with eight sampling locations of the zoantharians (This map was created using QGIS^®^ software under the terms of the GNU Free Documentation License Version 1.3 published by the Free Software Foundation. Copyright^©^ 2008 Free Software Foundation, Inc http://fsf.org).

Eight sites were sampled using SCUBA diving at the REMAPE (Fig. 5^63^ and see Supplementary Table S1).

### Biological material

A total of 36 zoantharians were mostly collected by SCUBA diving in different sites but very similar coralligenous habitats at depths between 2 m and 30 m around the “El Pelado” islet. The specimens of two species of *Zoanthus* were present in the intertidal zone (Fig. 1). The samples were collected between August 2015 and August 2016 at the Marine Protected Area “El Pelado” in the province of Santa Elena located off the mainland coast of Ecuador (Fig. 5). Samples of each species were split into three subsamples and fixed in: a) 4% formalin for morphological characterization, b) 95% ethanol for molecular analyses and c) frozen immediately after collection at −80 °C for metabolomics studies while waiting for freeze drying (250 mg fresh material at least were included in this last subsample). Only twelve specimens were analyzed for morphology and molecular data while six replicates for each species were required for the metabolomic approach (Table 2).

### Morphological examination

Morphological characters were examined from the formalin samples, *in situ* observations and *in situ* pictures. The data obtained included polyp measurements (oral disk diameter), number of tentacles, type of sphincter muscle, colors of the column, oral disk, tentacles and coenenchyme, relative amount of sand incrustation and host species or substrate (Table 1).

### DNA extraction and sequencing

DNA of zoantharian species were obtained from ethanol 95% preserved samples. DNA was extracted following the guanidine extraction protocol as described in Sinniger *et al*. 2010^64^. Samples were then amplified for COI and mt 16S r DNA using the primers LCOant, COIantr, 16Sant0a^64^ 16SbmoH^16^ following the thermal cycle conditions mentioned in Sinniger *et al*.^16,64^, the ITS rDNA using the primers Zoan-f, Zoan-r and the protocol described in Reimer *et al*.^30^ and 18S rDNA was amplified using the primers 18SA, 18SB^65^ and sequenced using 18SC, 18SL, 18SO, 18SY^66^ with standard Taq polymerase. The PCR amplification methods for each of the molecular markers were provided in the aforementioned references. The PCR amplified DNA fragments were sent to a commercial sequencing company (MacroGen Inc, Korea). New zoantharian sequences obtained in this study were deposited in GenBank, accessions numbers detailed for each specimen are reported in Table 2.

### Phylogenetic analyses

Each chromatogram was checked for quality using BioEdit software version 7.2.5.0. or Geneious 10.2.2 and trimmed appropriately. Multiple alignments were generated for each gene region from resulting FastA sequences, which were aligned by eye using sequences already available from previous work^25^. Available sequences from zoantharians were downloaded from GenBank and compared to the sequences generated here. Given the large number of identical sequences, only a subset of database sequences was included in phylogenetic reconstruction, and short sequences were avoided as much as possible. Details of database sequences used for comparison of similarity/identity or in the trees are identified in the results section. Given the variability in ITS rDNA sequences no phylogenetic tree was generated for this locus across all taxa. The CO1 alignment contained 50 sequences and was 807 bp in length. In the COI alignment, sequences of *Z*. *sansibaricus* KM267971 and KM267980 were truncated at 627 and 621 bp due to low confidence in the final bp of these sequences and the 16S sequence from the atlantic *Z*. *sociatus* from Iran LC164179 was assumed to be a misidentification of the Indo-Pacific *Z*. *sansibaricus*. The 16SrDNA alignment contained 91 sequences and was 1002 bp in length. Both alignments contained sequences of variable length leaving a significant number of gaps at each end. For the mitochondrial datasets maximum likelihood (ML) trees were obtained using PhyML 3.0 implemented in Geneious with a GTR model and estimated proportion of invariable sites, 6 substitution rate categories and estimated gamma distribution (as indicated as the optimal model by previous studies^10,67,68^). Bootstrap support was based on 100 iterations. Bayesian trees were obtained with MrBayes 3.1.2 implemented in Geneious. Four MCMC chains were run for 1.1 × 10^6^ generations under the model (GTR + Г + I) and with temperature of 0.2, burning of 1 × 10^5^ trees and thinning interval of 200. Both ML and Bayesian analyses were performed with gaps treated as missing data.

### Metabolomic analyses

To extract the metabolites of zoantharian species, six replicates of each species were collected from different depths and sites throughout the studied area (Fig. 5, See supplementary Table S1). Samples were frozen at −20 °C then freeze-dried and milled to yield a homogenous powder. Approximately 250 mg of powder were weighted and extracted three times with 5 mL of CH_2_Cl_2_/MeOH (1:1) in an ultrasonic bath for 5 min. The organic phases were combined and evaporated to dryness in a Speedvac (Thermo Scientific). Each extract was redissolved in 1 mL of a mixture of solvents CH_2_Cl_2_/MeOH (1:1) and placed in a 1.5 mL vial for UHPLC-HRMS analyses.

Mass spectra were obtained from an UHPLC–qToF (Agilent 1290) coupled to high resolution mass spectrometer (MS) equipped with an ESI source (Agilent 6540). LC/MS analysis were performed on positive mode, with an Acquity™ UPLC BEH C18 1.7 μm, 130 Å, 2.1 × 50 mm (Waters) column at a constant temperature of 40 °C. The mobile phase was prepared with: H_2_O + 0.1% formic acid (solvent A) and acetonitrile: H_2_O (95:5) + 0.1% formic acid (solvent B). Injection volume was set at 3 μL and elution rate at 0.4 mL.min^−1^. The elution gradient was programed as follow: 90% A – 10% B for 2 min, increasing B with a linear gradient up to 100% B from 2 to 10 min, isocratic gradient of 100% B during 2 min, return to the initial condition from 12 to 13 min, and 2 min of post-run for column equilibration, for a total runtime of 15 min. The mass spectrometer was calibrated with a formate/acetate solution before sample analysis. This calibration solution was automatically injected before each analysis for internal mass calibration. MS parameters were set as follow: nebulizer gas N_2_ at 40 psig, gas temperature: 300 °C, drying gas N_2_ at 4 L/min, ion source: ESI, TOF spectra acquisition from 50 to 1200 amu, capillary voltage: 3500 V. To randomize analytical sequences and reduce systematic error associated with instrumental drift, a Latin square was carried out. QC (pools) samples were analyzed at the beginning, the end and equidistantly throughout the sequence. Methanol (blank) samples were analyzed just before each QC sample to detect column contamination throughout the sequence.

LC–MS raw data files were converted to mzML files using the open-source msConvert tool from the ProteoWizard library, mzML files were processed using the R package XCMS (R version 3.3.1., XCMS version 1.50.0) to detect, deconvolute and align features (molecular entities with a unique and a specific retention time). Parameter settings for XCMS processing were set as described for UPLC-QTOF (high resolution)^69^. XCMS analysis of these data provided a matrix containing the retention time, *m/z* value and integrated peak area of the identified features. To filter analytical variation, only features with a coefficient of variation less than 20% among the QC samples were kept for further data analyses. Data were normalized by log transformation prior to statistical analysis. All these analyses are stored in Metabolights under the number MTBLS637.

Principal component analysis (PCA) was used to visualize the difference between the metabolomes of the different zoantharian species. Permutational multivariate analysis of variance (PERMANOVA, function adonis of the vegan package for R) and pairwise comparisons between group levels with corrections for multiple testing (function pairwise.perm.manova of the RVAideMemoire package for R) were used to evaluate statistically significant differences between the zoantharian species. A hierarchical cluster analysis (function hclust using Euclidian distance) was used to identify the similarities between the different zoantharian species. The ‘Average’ algorithm was chosen after analysis of the cophenetic correlation coefficients (Pearson correlation between the cophenetic distances calculated on cluster branches and the metabolite dissimilarity matrix). The Kelley-Gardner-Sutcliffe (KGS) penalty function was used to prune the dendrogram.

Metabolites with integrated areas equal or higher than 10^6^ in all samples of one species (considered the major metabolites observed in LC-MS) were selected. 45 out of 113 major metabolites were identified based on their exact mass and standards present in our laboratory. Kruskal-Wallis test and Kruskal post-hoc test was used to identify biomarkers (metabolites significantly overexpressed) in each of the zoantharian species.

## Electronic supplementary material

Supplementary Information
