# Supplementary material for: Assessing the Zoantharian Diversity of the Tropical Eastern Pacific through an Integrative Approach
Source: Sci Rep. 2018 May 8;8:7138. doi: 10.1038/s41598-018-25086-4 (PMC5940898; doi:10.1038/s41598-018-25086-4)
Supplement: Supplementary file 1 — Supplementary Information [file 41598_2018_25086_MOESM1_ESM.docx]

**Supplementary information**

**Assessing the Zoantharian diversity of the Tropical Eastern Pacific through an integrative approach**

**Karla B. Jaramillo^1, 2^, Miriam Reverter^3^, Paul O. Guillen^1, 3^, Grace McCormack^2^, Jenny Rodriguez^1^, Frédéric Sinniger^4^ *, and Olivier P. Thomas^3^ ***

^1^ ESPOL Polytechnic University, Escuela Superior Politécnica del Litoral, ESPOL. Centro Nacional de Acuicultura e Investigaciones Marinas, CENAIM. Campus Gustavo Galindo Km. 30.5 Vía Perimetral, P.O. Box 09-01-5863, Guayaquil, Ecuador.

^2^ National University of Ireland Galway, School of Zoology, Ryan Institute, University Road, H91 TK33 Galway, Ireland.

^3^ National University of Ireland Galway, School of Chemistry, Marine Biodiscovery, University Road, H91 TK33 Galway, Ireland.

^4^ Tropical Biosphere Research Center, University of the Ryukyus, Sesoko Island - Okinawa, 905-0227 Japan.

*Corresponding authors: [olivier.thomas@nuigalway.ie](mailto:olivier.thomas@nuigalway.ie), [fredsinniger@hotmail.com](mailto:fredsinniger@hotmail.com),

**Figure S1.** Phylogenetic Bayesian tree based on sequences of cytochrome c oxidase subunit I. Values at the nodes represent Bayesian bootstrap posterior probabilities of >0.75. and ML bootstrap probabilities (>75%) Values below posterior probabilities of 0.75 or 75% bootstrap were considered as unresolved. Specimens from this study in different colours.


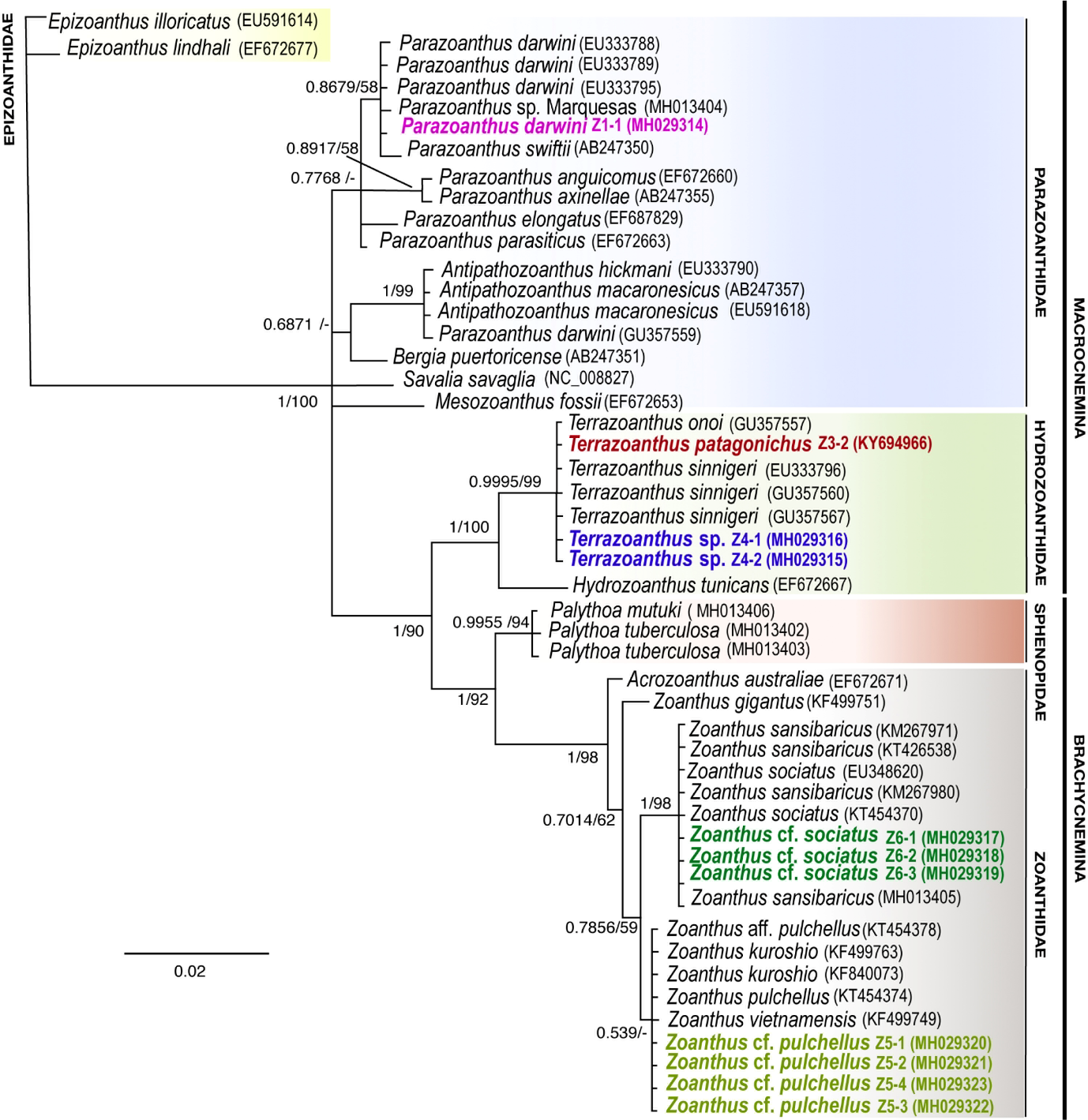


**
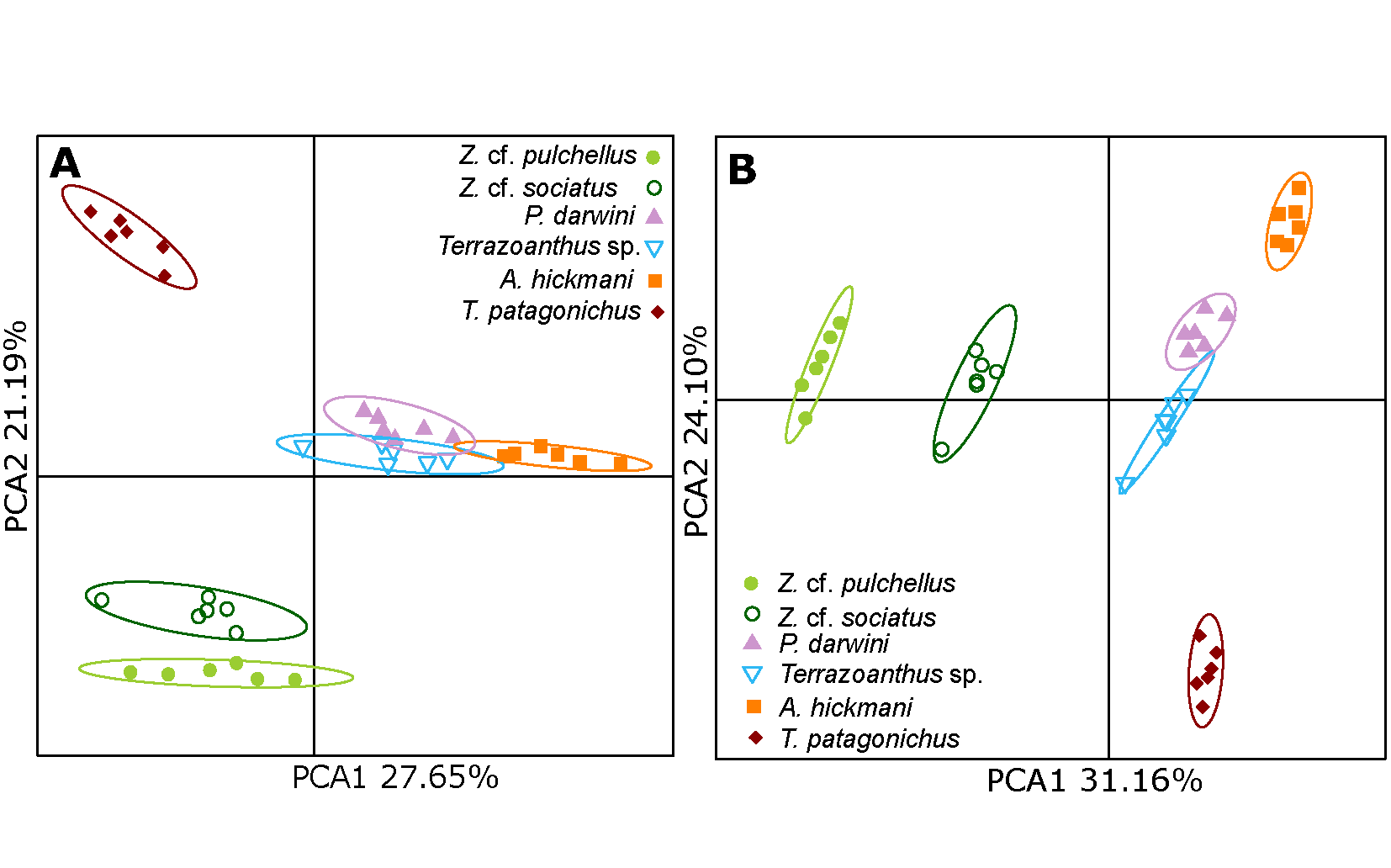
**

**Figure S2.** PCA of only major metabolites (areas ≥ 10^6^). The colours represent the different species of zoantharians.

**Table S1**. Geographical information on the sampling sites of the 6 replicates of the 6 Zoantharia species Z1 to Z6 across the REMAPE in the Province of Santa Elena- Ecuador.

| Sampling site  (Common name) | Samples | Latitude (S) | Longitude (W) | Depth (m) |
| --- | --- | --- | --- | --- |
| Pared | Z3-1 to -2  Z1-1 to -2  Z2-1 to -6 | 1°55’59.97’’ | 80°47’31.54’’ | 10-28 |
| Acuario | Z1-3 to -4 | 1°56’10.20’’ | 80°47’20.12’’ | 7-15 |
| Tello | Z3-3 to -4 | 1°55’35.10’’ | 80°47’16.28’’ | 26-33 |
| Laberinto | Z1-5 to -6  Z4-1 to -6 | 1°56’07.92’’ | 80°47’22.84’’ | 7-15 |
| Cuarenta | Z3-5 to -6 | 1°56’17.58’’ | 80°47’12.00’’ | 8-20 |
| Ayangue Bahía | Z5-1 to -3  Z6-1 to -2 | 1°58’53.04’’ | 80°45’20.69’’ | 0-3 |
| Ayangue Costa | Z5-1 to -3  Z6-3 to -4 | 1°59’02.80’’ | 80°45’35.41’’ | 3-5 |
| San Pedro Costa | Z6-5 to -6 | 1°57’52.62’’ | 80°44’16.24’’ | 0-1 |

**Metabolomic analyses**

*Parazoanthus darwini.*(Z1)

M215T55 (215.1051) : TrinorPseudozoanthoxanthin A (215.1051)

M229T86 (229.1210) : DiNorpseudozoanthoxanthin A (229.1157)

M306T137 (306.0365) : unknown

M203T160 (203.1556) : unknown

M384 386T217 (383.9474, 1 Br) : unknown

M432T228 (431.9331) : unknown

M481T243 (481.3174) : 20-hydroxyecdysone (481.3160)

M497T245 (497.3103) : Gerardiasterone (497.3109)

M281 283T252 (281.0664, 1 Br) : unknown

M212T259 (212.1773) unknown

M523T269 (523.3279), 20-hydroxyecdysone 2-acetate (523.3266)

M497T245 (497.3103) : unknown

M523T277 (523.3265) : 20-hydroxyecdysone 3-acetate (523.3266)

M509T281 (509.3127) : unknown

M465T304 (465.3223) : ecdysone (465.3211)

*Antipathozoanthus hickmani (Z2)*

M304T204 (304.1677), M330T209 (330.1834), M393 395 397T214 (393.0228, 1Cl 1Br), M437 439 441T220 (436.9724, 2Br), M463 465 467T224 (462.9880, 2Br), M485 487T230 (484.9586 1Br), M511 513T234 (510.9740, 1Br) : unknown

M481T243 (481.3178) : 20-hydroxyecdysone (481.3160)

M497T245 (497.3145) : Gerardiasterone (497.3109)

M537T259 (537.3077) : Ecdylactone A (537.3058)

M521T272 (521.3121) : Ecdylactone C (521.3109)

M523T269 (523.3283), 20-hydroxyecdysone 2-acetate (523.3266)

M523T277 (523.3265) : 20-hydroxyecdysone 3-acetate (523.3266)

M537T284 (537.3079) : Ecdylactone B (537.3058)

M523T292 (523.3283) : viticosterone (523.3266)

M465T304 (465.3221) : ecdysone (465.3211)

M521T325 (521.3124) : Ecdylactone D (521.3109)

M274T331 (274.2756) : unknown

M288T331 (288.2911) : unknown

M288T346 (288.2912) : unknown

*Terrazoanthus patagonichus.* (Z3)

M332T164 (332.1258) : dedimethylTerrazoanthine C

M393T211 (393.2056) : Terrazoanthine B (393.2043)

M407T221 (407.2210) : Terrazoanthine A (407.2187)

M346T227 (346.1459) : demethylTerrazoanthine C

M481T243 (481.3158) : 20-hydroxyecdysone (481.3160)

M340T244 (340.1905) : unknown

M360T250 (360.1568) : Terrazoanthine C (360.1553)

M523T269 (523.3271), 20-hydroxyecdysone 2-acetate (523.3266)

M523T277 (523.3265) : 20-hydroxyecdysone 3-acetate (523.3266)

M465T304 (465.3221) : ecdysone (465.3211)

*Terrazoanthus* sp*. (Z4)*

M481T243 (481.3171) : 20-hydroxyecdysone (481.3160)

M336T251 (336.2187) unknown

M212T259 (212.1773) unknown

M523T269 (523.3282), M523T277 (523.3308) : 20-hydroxyecdysone 2-acetate and 3-acetate (523.3266)

 M278T287 (278.2133) unknown

M465T304 (465.3221) : ecdysone (465.3211)

M274T330 (274.2753) : unknown

M288T340 (288.2909) : unknown

M288T345 (288.2909) : unknown

M376T380 (376.3266), M390T395 (390.3409), M404T410 (404.3574): unknown

*Zoanthus* cf. *pulchellus* (Z5)

M498T214 (498.2865) : 11-Hydroxynorzoanthamine (498.2850)

M512T230 (512.3020) : 11-Hydroxyzoanthamine (512.3007)

M512T236 (512.3024) : Oxyzoanthamine (512.3007)

M482T246 (482.2915) : Norzoanthamine (482.2900)

M496T251 (496.3080) : Zoanthamine (496.3057)

M496T254 (496.3080) : Zoanthamine (496.3057)

M554T263 (554.3126) : unknown

M516T272 (516.3000) : unknown

M490T319 (490.3395) : unknown

M274T330 (274.2753) : unknown

M288T340 (288.2909) : unknown

M376T385 (376.3222) : unknown

M404T408 (404.3537) : unknown

*Zoanthus* cf. *sociatus* (Z6)

M215T55 (215.1051) : trinorPseudozoanthoxanthin A (215.1051)

M229T78 (229.1207) : dinorpseudozoanthoxanthin

M243T151 (243.1365) : norpseudozoanthoxanthin

M257T218 (257.1528) : pseudozoanthoxanthin

M481T243 (481.3179) : 20-hydroxyecdysone (481.3160)

M523T269 (523.3269), 20-hydroxyecdysone 2-acetate (523.3266)

M523T277 (523.3265) : 20-hydroxyecdysone 3-acetate (523.3266)

M274T330 (274.2753) : unknown

M288T340 (288.2909) : unknown

M288T345 (288.2909) : unknown

M376T385 (376.3221) : unknown

M562T397 (562.3767) : unknown

M404T408 (404.3537) : unknown

M413T410 (413.3573) : unknown

M441T430 (441.3876) : unknown

M482T446 (482.3623) : unknown
